# Hard to “tune in”: neural mechanisms of live face-to-face interaction with high-functioning autistic spectrum disorder

**DOI:** 10.3389/fnhum.2012.00268

**Published:** 2012-09-27

**Authors:** Hiroki C. Tanabe, Hirotaka Kosaka, Daisuke N. Saito, Takahiko Koike, Masamichi J. Hayashi, Keise Izuma, Hidetsugu Komeda, Makoto Ishitobi, Masao Omori, Toshio Munesue, Hidehiko Okazawa, Yuji Wada, Norihiro Sadato

**Affiliations:** ^1^Department of Cerebral Research, National Institute for Physiological SciencesOkazaki, Aichi, Japan; ^2^Department of Physiological Sciences, The Graduate University for Advanced StudiesOkazaki, Aichi, Japan; ^3^Graduate School of Environmental Studies, Nagoya UniversityNagoya, Japan; ^4^Research Center for Child Mental Development, University of FukuiEiheiji, Fukui, Japan; ^5^Department of Neuropsychiatry, Faculty of Medical Sciences, University of FukuiEiheiji, Fukui, Japan; ^6^Biomedical Imaging Research Center, University of FukuiEiheiji, Fukui, Japan; ^7^Faculty of Nursing and Social Welfare Sciences, Fukui Prefectural UniversityEiheiji, Fukui, Japan; ^8^Research Center for Child Mental Development, Kanazawa UniversityKanazawa, Ishikawa, Japan

**Keywords:** functional connectivity, hyperscanning, inter-subject coherence, joint attention, mutual gaze, autistic spectrum disorder, functional magnetic resonance imaging

## Abstract

Persons with autism spectrum disorders (ASD) are known to have difficulty in eye contact (EC). This may make it difficult for their partners during face to face communication with them. To elucidate the neural substrates of live inter-subject interaction of ASD patients and normal subjects, we conducted hyper-scanning functional MRI with 21 subjects with autistic spectrum disorder (ASD) paired with typically-developed (normal) subjects, and with 19 pairs of normal subjects as a control. Baseline EC was maintained while subjects performed real-time joint-attention task. The task-related effects were modeled out, and inter-individual correlation analysis was performed on the residual time-course data. ASD–Normal pairs were less accurate at detecting gaze direction than Normal–Normal pairs. Performance was impaired both in ASD subjects and in their normal partners. The left occipital pole (OP) activation by gaze processing was reduced in ASD subjects, suggesting that deterioration of eye-cue detection in ASD is related to impairment of early visual processing of gaze. On the other hand, their normal partners showed greater activity in the bilateral occipital cortex and the right prefrontal area, indicating a compensatory workload. Inter-brain coherence in the right IFG that was observed in the Normal-Normal pairs (Saito et al., 2010) during EC diminished in ASD–Normal pairs. Intra-brain functional connectivity between the right IFG and right superior temporal sulcus (STS) in normal subjects paired with ASD subjects was reduced compared with in Normal–Normal pairs. This functional connectivity was positively correlated with performance of the normal partners on the eye-cue detection. Considering the integrative role of the right STS in gaze processing, inter-subject synchronization during EC may be a prerequisite for eye cue detection by the normal partner.

## Introduction

Autistic spectrum disorder (ASD) encompasses both autism and Asperger syndrome (Wing et al., 2002). Previous research has addressed the epidemiology of these increasingly prevalent disorders (Baird et al., 2006). Individuals with ASD have core impairments in reciprocal social interactions, abnormal development and use of language, repetitive and ritualized behaviors, and a narrow range of interests (Kanner, 1943; Asperger, 1944). The etiology of ASD remains largely unknown. Impairment of social attention such as joint attention and eye contact (EC) is regarded as an early sign of ASD (Baron-Cohen, 2008).

Joint attention refers to the ability to “coordinate attention between interactive social partners with respect to objects or events in order to share an awareness of the objects or events” (Mundy et al., 1986). It emerges as early as 6–12 months of age (Corkum and Moore, 1998). Two types of joint attention behavior emerge in the first months of life: Responding to Joint Attention (RJA) refers to infants' ability to follow the direction of gaze. Initiating Joint Attention (IJA) refers to infants' ability to spontaneously create a shared point of reference by the use of alternating gaze between objects and other people with EC (Mundy et al., 2009).

EC is implicated in the sharing of various psychological states such as intention (Searle, 2001), attention, and emotion (Trevarthen, 1979; Hobson, 2002), making inter-subjectivity possible. An adult's initial EC prior to looking at an object is a critical cue that can establish joint attention with infants as young as 9 months old (Striano et al., 2006). EC might therefore provide a communicative context for joint attention (Farroni et al., 2002). This is an example of the “eye-contact effect,” which is defined as a phenomenon in which perceived EC modulates the concurrent and/or immediately following cognitive processes and/or behavioral responses (Senju and Johnson, 2009).

Individuals with ASD show unusual patterns of joint attention (Mundy et al., 2009) and eye-contact behavior (Volkmar and Mayes, 1990; Buitelaar, 1995). Joint attention disabilities have been posited to be a pivotal deficit in autism (Osterling and Dawson, 1994; Mundy and Crowson, 1997; Charman, 2003). IJA is a better diagnostic discriminator of autism than is RJA (Mundy et al., 1986; Sigman and Ruskin, 1999; Lord et al., 2000; Charman, 2003; Dawson et al., 2004; Hobson and Hobson, 2007). In particular, diminished alternating EC to share attention with respect to object is an important measure of IJA impairment in autism (Mundy et al., 2009). EC is not used to initiate joint attention by individuals with ASD (Sigman et al., 1986; Baron-Cohen, 1989, 1995). Senju et al. (2003) found that children with autism were no better at detecting direct gaze than averted gaze, whereas typically-developing children were more efficient at detecting the former; their study also suggested that a lack of ability to detect direct gaze might result in altered eye-contact behavior, which in turn could hamper the subsequent development of social skills.

There are several neuroimaging studies to depict the neural substrates of IJA and RJA. Williams et al. (2005) conducted RJA task that focused on the sharing the attention towards the objects. In the joint attention condition, the model's gaze and the dot movement was concordant whereas that was discordant in non-joint attention condition. Activated area is in the anterior and posterior cingulate cortices. Another important characteristic of joint attention is the liveness. Using live interaction joint attention tasks, Redcay et al. (2010, 2012a) depicted activation patterns of IJA and RJA in normal control group. Distinct regions included the ventromedial prefrontal cortex for RJA and intraparietal sulcus and middle frontal gyrus for IJA. Overlap was observed in the dorsal medial prefrontal cortex (dMPFC), right inferior frontal gyrus (IFG), and right posterior superior temporal sulcus (pSTS) for IJA and RJA. Utilizing virtual reality technique and functional magnetic resonance imaging (fMRI), Schilbach et al. (2010) showed that IJA and RJA reflected activation of independent neural networks. They found unique activation for IJA in the ventral striatum bilaterally, and activation of the ventral medial prefrontal cortex for RJA.

Neural substrates of eye gaze have been studied extensively, highlighting the importance of the pSTS (for review, see Frischen et al., 2007). Bilateral removal of the STS region in macaques produces impaired perception of gaze direction without significantly affecting facial identity perception (Heywood and Cowey, 1992). Recent human fMRI studies have identified the involvement of the pSTS in social perception through eye movement (Allison et al., 2000), including EC (Calder et al., 2002; Wicker et al., 2003; Pelphrey et al., 2005). Gaze processing extends to include the amygdala (Kawashima et al., 1999; George et al., 2001), the inferior temporal (Wicker et al., 1998), parietal (Wicker et al., 1998; Hoffman and Haxby, 2000; Hooker et al., 2003; Mosconi et al., 2005; Calder et al., 2007), medial prefrontal, and anterior cingulate cortices (Calder et al., 2002; Williams et al., 2005), and other frontal regions (Hooker et al., 2003; Mosconi et al., 2005; Williams et al., 2005; Bristow et al., 2007). These different regions seem to process different aspects of the visual and social properties of gaze.

These previous works on the neural substrates of social attention have been conducted with single-participant fMRI. Thus, the eye-contact related activation may not represent the pair-specific psychological state, or inter-subjective sharing, that was established by the EC of two persons engaged in actual EC and joint attention. To depict pair-specific neural activities, Saito et al. (2010) conducted an RJA and mutual gaze paradigm using dual fMRI (Saito et al., 2010) with a hyperscanning method (Montague et al., 2002). During an RJA task in which two participants were scanned simultaneously by fMRI, the eye-cued task activated the bilateral occipital pole (OP) extending to the right pSTS, the dMPFC, and the bilateral IFG. An interaction between eye movement and shared attention towards an object was found in the left intraparietal sulcus. After the task-related effects were modeled out, inter-individual correlation analysis was performed on the residual time-course data. Paired subjects showed more prominent correlations than non-paired subjects in the right IFG, suggesting that this region is involved in shared intention during EC, which provides the context for RJA (Saito et al., 2010). These results indicate that both eye-contact and eye-gaze detection are important for RJA, and that pair-specific neural synchronization in the right IFG during EC may represent the psychological common ground between two person with EC.

However, it remains unclear which processes of the social attention are impaired in ASD and their neural substrates, particularly when they are confronted with the partners in the live, real-time face-to-face interaction.

The present study investigated the neural representation of social attention in individuals with ASD during a face-to-face live interaction with normal partner. We hypothesized that ASD individuals would establish less shared intention by EC, which might lead to reduced performance of RJA task. Specifically, we expected ASD–Normal pairs to show less inter-individual synchronization in the right IFG during EC than that previously reported for Normal–Normal pairs (Saito et al., 2010). As shared intention represents the psychological common ground with a partner, we anticipated that the performance and neural activity of an ASD participant's normal partner would also be affected.

Here we compared the neural substrates, inter-individual functional connectivity, and default mode network activity during EC and joint attention between ASD participants and normal participants using dual fMRI, following our previously reported protocol (Saito et al., 2010). We recruited 21 pairs of participants with high-functioning ASD and age- and sex-matched normal control adults, and 19 pairs of normal participants (Saito et al., 2010). During the experiment, EC was maintained at baseline while the subjects engaged in real-time gaze exchange in an RJA task. The task-related effects were modeled out, and the correlation between the two subject's brain activities was calculated using the residual time-series data for each voxel (inter-individual correlation analysis). If the inter-subject coherent activity in the right IFG represents the common psychological ground (Saito et al., 2010) that modulates cognitive processes, intra-subject functional connectivity in the right IFG might represent the effect of EC. Therefore, the intra-brain default-mode network was evaluated with the right IFG as a seed region, in order to visualize the targets of the eye-contact effect. Specifically, we expected areas involved in higher-level eye-gaze processing, such as the STS, to show functional connectivity with the right IFG, the strength of which should depend on performance.

## Materials and methods

### Participants

Sixteen males and five females with high-functioning ASD [mean age ± standard deviation (SD) = 25.1 ± 5.3 years; age range = 17 – 39 years] were recruited at the Department of Neuropsychiatry of the University of Fukui Hospital, and the Department of Psychiatry and Neurobiology of the Kanazawa University Hospital, in Japan.

The authors (Hirotaka Kosaka and Toshio Munesue) diagnosed the participants based on the classifications described in the Diagnostic and Statistical Manual of Mental Disorders (DSM-IV-TR; American Psychiatric Association, 2000) and on standardized criteria taken from the Diagnostic Interview for Social and Communication Disorders (DISCO; Wing et al., 2002); these authors were trained in the diagnosis of ASD under Dr. Tokio Uchiyama, and were qualified to use the DISCO Japanese edition. The DISCO has good psychometric properties (Nygren et al., 2009). It contains items on early development, and a section on activities of daily life, thereby giving the interviewer an idea of the level of functioning in several different areas, not only social functioning and communication (Wing et al., 2002). In the ASD group, sixteen participants were diagnosed with autism and five with Asperger syndrome.

We also recruited 21 age-and sex-matched typically developed (normal) individuals (16 males and five females; mean age ± SD = 24.0 ± 3.7 years; age range = 19–31 years) from the local community. Participants were excluded if they had a history of major medical or neurological illness including epilepsy, significant head trauma, or a lifetime history of alcohol or drug dependence. They were screened to exclude individuals who had a first-degree relative with an axis I disorder, based on the DSM-IV criteria. In addition, we employed the previous data from 19 pairs of normal participants (Saito et al., 2010) as controlled Normal groups [19 males, mean age ± SD = 23.8 ± 4.0 years for the Normal group (3T); 19 males, mean age ± SD = 25.6 ± 4.8 years for the Normal group (1.5T)], to avoid machine effect and interaction effect with ASD during the interactive situation such as mutual gaze and joint attention.

To check the difference of the general ability, we carried out Intelligence Quotient (IQ) assessments using the Wechsler Adult Intelligence Scale-III (WAIS-III; Wechsler, 1997) for ASD and Normal paired with ASD groups. All of the participants had full-scale IQ scores >80, although average of IQ scores in Normal was higher than that in ASD (mean ± SD = 101.2 ± 16.2 for the ASD, 113.7 ± 6.1 for the Normal paired with ASD, *t* = 3.04, *p* < 0.01). According to the Normal groups from the previous study, we were not able to perform IQ assessment.

We also measured autistic traits using the autism-spectrum quotient (AQ) (Baron-Cohen et al., 2001) for all four groups [i.e., ASD, Normal paired with ASD, Normal (3T), Normal (1.5T)]. AQ scores of ASD group were significantly higher compared to those of other three Normal groups [mean ± SD = 29.9 ± 7.3 for ASD, 17.0 ± 6.0 for Normal paired with ASD, 19.9 ± 6.5 for Normal (3T), 19.9 ± 5.2 for Normal (1.5T); analysis of variance (ANOVA), *F*_(3, 78)_ = 16.04, *p* < 0.001; *post-hoc t*-test with Bonferroni correction, *p* < 0.001 in ASD vs. Normal paired with ASD, *p* < 0.001 in ASD vs. Normal (3T), *p* < 0.001 in ASD vs. Normal (1.5T), respectively], and there were no statistically differences among three Normal groups [*post-hoc t*-test with Bonferroni correction, *p* = 0.94 in Normal paired with ASD vs. Normal (3T), *p* = 0.94 in Normal paired with ASD vs. Normal (1.5T), *p* = 1.00 in Normal (3T) vs. Normal (1.5T), respectively]. The detailed demographic data and scores were shown in Table 1.

**Table 1:** **(R1-1) Demographic data and rating scale scores**.

**Experiment**	**ASD–Normal Exp.**		**Normal–Normal Exp.**	
**Participants**	**ASD (3T)**	**Normal (1.5T)**	**Normal (3T)**	**Normal (1.5T)**
Number (male/female)	16/5	16/5	19/0	19/0
Handedness ^*a*^ (right/left)	21/0	21/0	19/0	19/0
Age at examination	25.1 ± 5.3	24.0 ± 3.7	23.8 ± 3.5	25.6 ± 4.8
WAIS-III: full-scale IQ	101.2 ± 16.2	113.7 ± 6.1	n.a.	n.a.
WAIS-III: verbal IQ	107.3 ± 16.2	116.0 ± 7.5	n.a.	n.a.
WAIS-III: performance IQ	93.5 ± 16.5	107.4 ± 8.2	n.a.	n.a.
AQ: total score	29.9 ± 7.3	17.0 ± 6.0	19.9 ± 6.5	19.9 ± 5.2
AQ: social skill scores	6.5 ± 2.9	2.9 ± 1.8	2.8 ± 2.4	2.9 ± 2.4
AQ: attention-switching scores	6.5 ± 1.6	4.4 ± 2.3	5.2 ± 2.0	4.9 ± 1.6
AQ: attention-to-detail scores	6.3 ± 2.9	4.0 ± 2.4	4.9 ± 2.1	5.2 ± 2.1
AQ: communication scores	5.7 ± 2.9	3.0 ± 2.2	3.2 ± 2.5	3.6 ± 2.2
AQ: imagination scores	5.0 ± 2.4	2.8 ± 1.5	3.8 ± 2.0	3.2 ± 1.9

Values are given as mean ± SD. AQ, Autism Spectrum Quotient (Baron-Cohen et al., 2001); ASD, autism spectrum disorder; n.a., not available; Normal, typically-developed; WAIS-III, Wechsler Adult Intelligence Scale Third Edition; 1.5T, participants who set in the 1.5T MR scanner; 3T, participants who set in the 3T MR scanner;

a According to the Edinburgh handedness inventory (Oldfield, 1971).

The protocol used for the present study was approved by the Ethical Committee of the University of Fukui. After a complete explanation of the study, all of the participants gave written informed consent prior to the experiment.

### Experimental setting

#### Hardware

The experimental setting and task procedure were the same as in our previous study (Saito et al., 2010). Briefly, brain activity was recorded while paired subjects in two MRI scanners performed an online gaze-exchange task. An infrared face-recording and eye-tracking system (NAC Image Technology Inc., Tokyo, Japan) was used to combine the two MRI systems. Video images of participants' faces were recorded by an infrared camera and transferred to a personal computer (Dimension 9200; Dell Computer, Round Rock, TX, USA). The visual stimuli (ball targets) were presented using Presentation software (Neurobehavioral Systems, Albany, CA, USA). Images of participants' eyes and eyebrows were combined with the visual stimuli using a screen splitter (MV-40F; FOR-A, Tokyo, Japan), and transmitted using a liquid crystal-display projector (TH-AE900; Panasonic Co., Osaka, Japan) onto a half-transparent screen positioned on top of a 3 Tesla (3T) or 1.5T MRI scanner bed approximately 255 cm or 304 cm, respectively, from the participants' eyes. The visual angle of the screen was 7.1 × 10.4°. There was no image delay between actual eye movement and the presentation of it to the partner.

**Figure 1:**
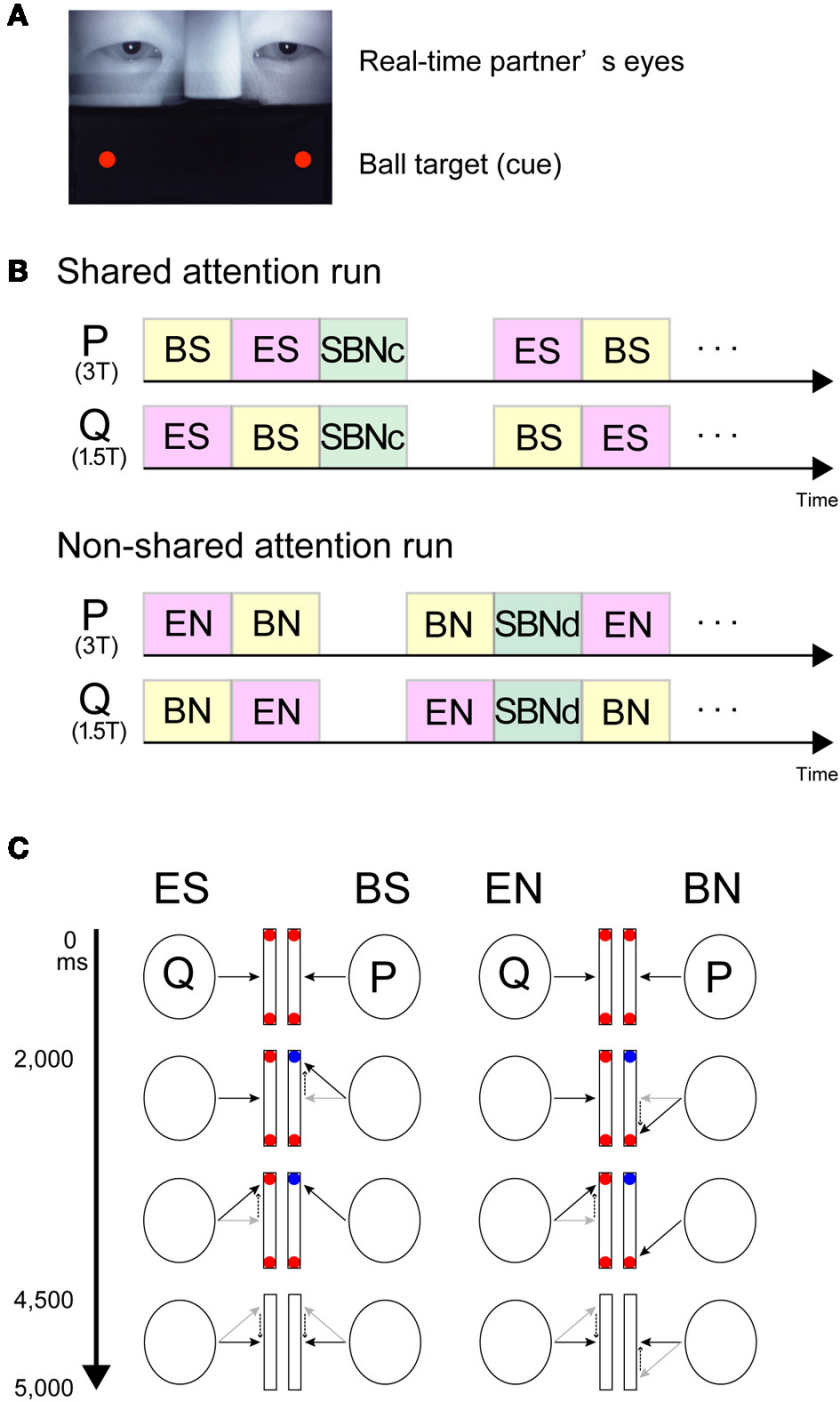
**Visual stimuli and schematic task diagram. (A)** Screen snapshot showing real-time images of the partner's eyes (upper part) combined with the ball cues generated by the stimulus presentation software (lower part). **(B)** Stream of the experiment. Participants (P and Q) were paired and placed in a 3T or 1.5T MRI scanner, respectively. Upper part, example of the shared attention run. Lower part, example of the non-shared attention run. **(C)** Schematic diagram of the joint-attention task. Participants (P and Q) were paired and placed in a 3T or 1.5T MRI scanner, respectively. Black arrows indicate gaze direction. Dotted arrows demonstrate a gaze shift from the grey to black arrows. Red and blue balls indicate the cues on screen. In the ES and BS conditions, the participants were required to shift their gaze to the target cued by either the partner's gaze (ES) or the color-change of a ball (BS). Each task lasted 5 s. In the EN and BN conditions, the participants were required to shift their gaze to the opposite side of the target. The condition was switched according to the task. ES, eye-cued, shared attention condition; BS, ball-cued, shared attention condition; SBNc, simultaneous ball-cued and not shared attention condition during concordant (shared-attention) run; EN, eye-cued, non-shared attention condition; BS, ball-cued, non-shared attention condition; SBNd, simultaneous ball-cued and not shared attention condition during discordant (non-shared attention) run.

In the MRI scanner, each participant performed the joint-attention task while engaging in real-time gaze exchange. Images of their partner's eyes were presented on the upper part of the screen, and images of two balls were presented on both sides of the lower part of the screen (Figure 1A).

#### Experimental design and task procedures

The task was to look at one of the ball targets cued either by the eye movement of the partner or by the change in color of the ball target (Saito et al., 2010).

There were two types of runs depending on the cue-response behavior. During concordant runs (Figure 1B left), participants were required to shift their gaze to the cued target. During discordant runs (Figure 1B right), participants were asked to shift their gaze to the opposite side to where the target appeared. Explicit instructions were given to both subjects at the start of each run.

In concordant runs, four tasks were configured by three types of the ball cue presentation. As the first type (Figure 1B left), the ball cue was provided to one participant. Here, following EC for 2000 ms with two red balls in the lower half of the screen, one of the balls in front of one participant (say, P) changed to blue for 2500 ms. The participant P was required to shift his gaze to the changed ball as soon as possible. The counterpart (say, Q) was asked to gaze at the ball (which from his or her perspective does not change in color) that P attended to. Then, the balls on both sides disappeared for 500 ms, at which point the participants returned to joint EC. As participants P and Q watched the same ball target, P underwent ball-cued shared attention [ball-share (BS)] and Q underwent eye-cued shared attention [eye-share (ES)]. As the second type, the ball cue was provided to both participants simultaneously (not shown in Figure 1). In this case, following EC for 2000 ms with two red balls in the lower half of the screen, one of the balls in front of both participants changed to blue simultaneously, but on different sides, for 2500 ms. The participants were required to shift their gaze to the changed ball. Thus, both participants underwent simultaneously ball-cued non-shared attention [simultaneous ball-non-share during concordant run (SBNc)]. As the third type, no ball cue was provided on either side. EC trials started with EC without any ball cue; thus, the participants continued to hold EC for 4500 ms, followed by the balls disappearing for 500 ms (not shown in Figure 1).

During discordant runs (Figure 1B right), the participants were asked to shift their gaze to the opposite side where the target appeared. The set-up was identical to the concordant runs. Thus, when the ball cue was provided to one side, P underwent ball-cued non-shared attention [ball-non-share (BN)], and Q underwent gaze-cued non-shared attention [eye-non-share (EN)]. When the ball cue was provided to both sides, both participants simultaneously underwent ball-cued non-sharing attention [simultaneous ball-non-share during discordant run (SBNd)].

The four task conditions, ES and BS during concordant runs, and EN and BN during discordant runs, were contrasted with each control condition (SBNc for concordant runs and SBNd for discordant runs) to generate contrast images of ES', BS', EN', and BN' respectively, which in turn constituted a 2 (cue, eye vs. ball) × 2 (attention, sharing vs. non-sharing) design. A schematic diagram of the task is shown in Figure 1C. To reduce the participant's workload, the same condition was repeated three times in one block (15 s).

### MRI data acquisition

All images were acquired using a 3T or 1.5T MRI scanner (Signa Exite; General Electric, Milwaukee, WI, USA) with an eight-element phased-array coil. For functional images, we used an interleaved T2^*^-weighted gradient-echo echo-planar imaging (EPI) technique to obtain 85 volumes of time-series image data. Each volume consisted of 34 continuous 4-mm-thick slices with no gap, in order to cover the entire cerebral cortex and cerebellum [repetition time (TR) = 3000 ms; echo time (TE) = 30 ms for 3T and 45 ms for 1.5T; flip angle (FA) = 90°; field of view (FOV) = 192 mm; 64 × 64 in-plane matrix]. The head motion was minimized by placing soft spacers between each participant's head and the coil. Three-dimensional (3D) spoiled-gradient recalled-echo (SPGR) images (TR = 33 ms; TE = 3.0 ms; FA = 30°; FOV = 240 mm; matrix size = 256 × 192 pixels; slice thickness = 1.5 mm; a total of 112 transaxial images) were obtained in order to acquire a high-resolution structural image of the whole brain. To minimize the task-induced signal change caused by differences in magnetic strength, a longer TE was used for the 1.5T scanner (45 ms) than the 3T scanner (30 ms), based on a preliminary fMRI experiment with visual checkerboard stimuli (Saito et al., 2010).

### Image preprocessing

All of the data used in the present study, and those acquired in our previous study (Saito et al., 2010), were analyzed using Statistical Parametric Mapping software version 8 (SPM8; Wellcome Department of Imaging Neuroscience, London, UK) implemented in MATLAB 2010a (MathWorks, Natick, MA, USA).

The first five volumes of each run were eliminated to allow for stabilization of the magnetization, and the remaining 80 volumes per run (a total of 480 volumes per participant) were used for the analysis. After correcting for differences in slice timing within each image volume, all of the volumes were realigned for motion correction. The sixth EPI volume was normalized to the Montréal Neurological Institute (MNI) EPI template, and the same parameters were applied to all of the other EPI volumes. They were then spatially smoothed in three dimensions using an 8 mm full-width-at-half-maximum Gaussian kernel.

### Statistical analysis

To depict the neural substrates of the tasks, we adopted a summary statistics approach. First, the task-related activity in each individual was modeled as regressors convolved with a canonical hemodynamic response function (HRF). The data were high-pass filtered with a cut-off period of 128 s to remove low-frequency signal drifts. A first-order autoregressive model [AR(1)] was used to remove serial correlations in the signals (Friston et al., 2007). The parameters were estimated using the general linear model. To test the hypothesis about condition effects, the estimates for each of the model parameters were compared with the linear contrasts. The contrast images, the weighted sum of the parameter estimates, were used for the second-level analysis with a random-effects model, in order to make inferences at the population level (Friston et al., 1999).

We employed a 2 × 2 × 2 factorial design to detect the main effects of group (ASD 3T vs. Normal paired with ASD 1.5T or Normal 3T vs. Normal 1.5T), cue (Eye vs. Ball), and attention (Shared vs. Non-shared), and their interactions. For the group factor, the four cells were constructed by the contrast images of ES–SBNc, BS–SBNc, EN–SBNd, and BN–SBNd. To simplify the notation, these were labeled as ES', BS', EN', and BN', respectively. To depict the neural substrates of the specific interaction of ASD–Normal pairs, we directly compared the ASD–Normal and Normal–Normal groups from our previous study (Saito et al., 2010) in a second-level analysis. To eliminate the scanner effect (3T vs. 1.5T), we compared the ASD (3T) and Normal groups from the same MRI scanner (3T). We also compared the normal participants paired with ASD participants with the normal participants whose data were acquired in the same scanner (1.5T). The resulting set of voxel values for each contrast constituted a statistical parametric map of the *t* statistic (SPM{*t*}). The threshold for the SPM{*t*} was set at *p* < 0.05 with a family-wise error (FWE) correction at the cluster level for the entire brain (Friston et al., 1996), unless otherwise indicated. We used the same analytical approach in our previous study (Saito et al., 2010).

### Estimate of functional connectivity between participants

To subtract the effect of the task-related activity, all of the conditions were modeled and estimated using a general linear model (Villalobos et al., 2005; Fair et al., 2007). In its standard form, SPM8 does not save the residuals at each volume. We therefore modified the program spm_spm.m to obtain the residuals, and concatenated the residuals with all of the runs. The first two residual time-points of each run were discarded. Correlation of the residuals between the same coordinate positions of two (normalized) brains was calculated for every voxel. The correlation *r* value was transformed to a *z*-score using Fisher's *r*-to-*z* transformation, and images containing the *z*-scores of every voxel were generated. All possible combinations of the pairs (21 × 21 = 441 pairs in the ASD–Normal experiment; 19 × 19 = 361 pairs in the Normal–Normal experiment) were generated and divided into four groups as follows: 21 combinations in which one ASD and one normal individual participated in the experiment simultaneously (Pair in ASD–Normal experiment); 420 combinations in which they did not (Non-pair in ASD–Normal experiment); 19 combinations in which two normal subjects participated in the experiment simultaneously (Pair in Normal–Normal experiment); and 342 combinations in which they did not (Non-pair in Normal–Normal experiment). The residual data from our previous study (Saito et al., 2010) were obtained with SPM5, so that we reanalyzed all the Normal–Normal experimental data with SPM8. As we were concerned with the difference between Pair and Non-pair and compared of them, scanner effect (i.e., the difference caused by the MR scanners) was not contaminated.

### Estimate of within-brain functional connectivity

To investigate whether intra-individual functional connectivity involving the right IFG differed across groups, we conducted region of interest (ROI)-to-voxel functional connectivity analysis (Biswal et al., 1995). Additional preprocessing procedures were performed with CONN (http://www.nitrc.org/projects/conn). We introduced 0.01–0.06-Hz band-pass temporal filtering to remove magnetic field drifts of the scanner (Foerster et al., 2005) and physiological noise components falling in high-frequency bands (Cordes et al., 2001). We applied the functional connectivity analysis to the residual time-series data, as in our previous study (Saito et al., 2010). Upon checking the residual data, we recognized that the task effect was not completely removed from the raw data even after applying the AR model implemented in SPM8 (Friston et al., 2007); it was still evident around 0.067 Hz, consistent with our previous results (Saito et al., 2010). To remove the influence of the task effect on the estimation of functional connectivity in the eye-contact condition, the cut-off frequency (0.06 Hz) was set at a lower value than that used in standard functional connectivity analysis. For the inter-individual correlations, the seed region was defined using the group data analysis, with a statistical threshold of *p* < 0.05 and a FWE correction at the cluster level for the entire brain. The residual time-series data within the ROI were then averaged individually, and used as the right ventral IFG time-course data. After preprocessing, a voxel-wise correlation map was calculated for each individual using the CONN program. The correlation maps were converted to *z*-values using Fisher's *r*-to-*z* transformation to enable group-level comparisons. Voxel-wise group analyses of the correlation maps were performed with two-sample *t*-tests using SPM8. The statistical threshold was set at *p* < 0.05 with an FWE correction at the cluster level for the entire brain.

## Results

### Behavioral results

**Figure 2:**
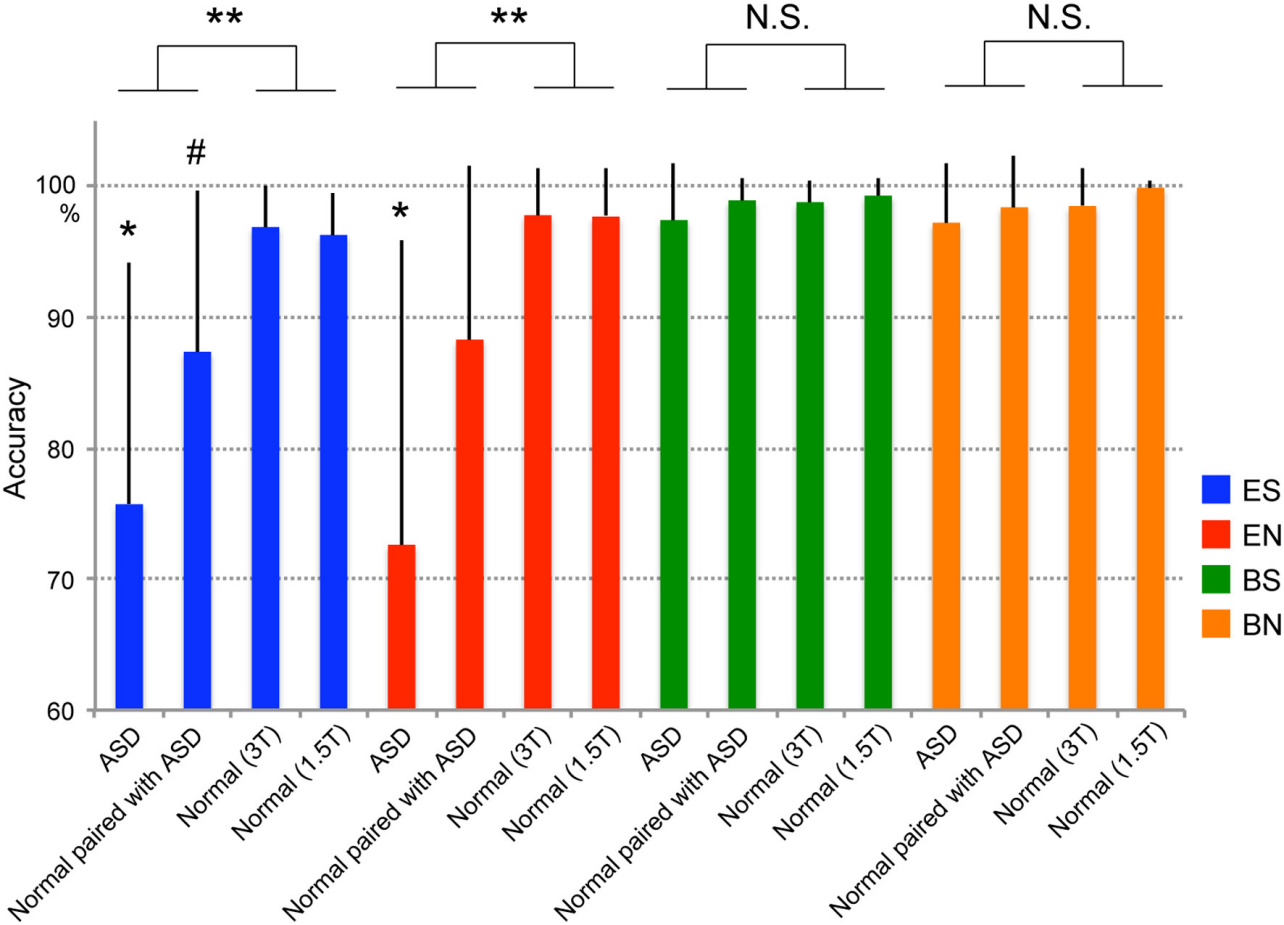
**Task performance (accuracy).** Blue indicates the ES condition (eye cued and shared attention), red the EN condition (eye cued and non-shared attention), green the BS condition (ball cued and shared attention), and orange the BN condition (ball cued and non-shared attention). ASD denotes individuals with autism spectrum disorder. Paired with ASD denotes normal individuals who were paired with ASD participants during the experiment. Normal (3T) and Normal (1.5T) denote normal individuals who were paired with normal individuals in 3T or 1.5T MRI scanners, respectively, during the experiment. Error bars indicate the standard error of the mean (SEM). Statistically significant differences were observed between ASD–Normal and Normal–Normal pairs in the ES and EN conditions (^**^p < 0.001), but not in the BS and BN conditions [not significant (N.S.)]. ^*^Statistical difference (*p* < 0.01) between the ASD group and the other three groups in the ES and EN conditions. #Statistical trend between ASD and Normal (3T) groups (*p* = 0.065) or between the ASD and Normal (1.5T) groups (*p* = 0.099) in the ES condition.

To compare the differences between ASD participants, Normal participants paired with ASD participants, and Normal participants, we conducted a Two-Way repeated-measures analysis of variance (rmANOVA) incorporating Group (ASD at 3T, Normal paired with ASD at 1.5T, Normal at 3T, Normal at 1.5T) and Task (ES, EN, BS, and BN) (Figure 2). All participants were not informed who his/her partner was, and nobody in Normal group was aware from his/her partner's behaviors that partner had social impairments. A Group × Task interaction was observed [*F*_(4.548, 115.225)_ = 12.040 with Greenhouse–Geisser correction, *p* < 0.001]. To assess this finding, we then tested for the main effect of Group across the Tasks. The results showed that the difference among the four groups was observed only in the eye-cued conditions such as ES [*F*_(1, 79)_ = 30.591, *p* < 0.001] and EN [*F*_(1, 79)_ = 26.707, *p* < 0.001], and not in the ball-cued conditions such as BS [*F*_(1, 79)_ = 2.235, *p* = 0.139] and BN [*F*_(1, 79)_ = 3.365, *p* = 0.070]. *Post hoc* tests with the Bonferroni correction showed that the ASD group was significantly less accurate than the other three groups in the ES (ASD vs. Normal paired with ASD, *p* < 0.01; ASD vs. Normal 3T, *p* < 0.001; ASD vs. Normal 1.5T, *p* < 0.001) and EN (ASD vs. Normal paired with ASD, *p* < 0.005; ASD vs. Normal 3T, *p* < 0.001; ASD vs. Normal 1.5T, *p* < 0.001) conditions. The Normal participants paired with the ASD subjects also tended to show lower accuracy than the Normal groups (Normal paired with ASD vs. Normal 3T, *p* = 0.065; Normal paired with ASD vs. Normal 1.5T, *p* = 0.099), although these differences did not reach statistical significance. By contrast, there were no differences in any combination of the groups in the ball-cued conditions (BS and BN). (RR1-1) There was no significant correlation between IQ and performance of each condition (IQ and ES, *r* = 0.198, *p* = 0.415; EN, *r* = 0.322, *p* = 0.179; BS, *r* = 0.205, *p* = 0.400; BN, *r* = 0.308, *p* = 0.199, respectively).

### Activation results

**Figure 3:**
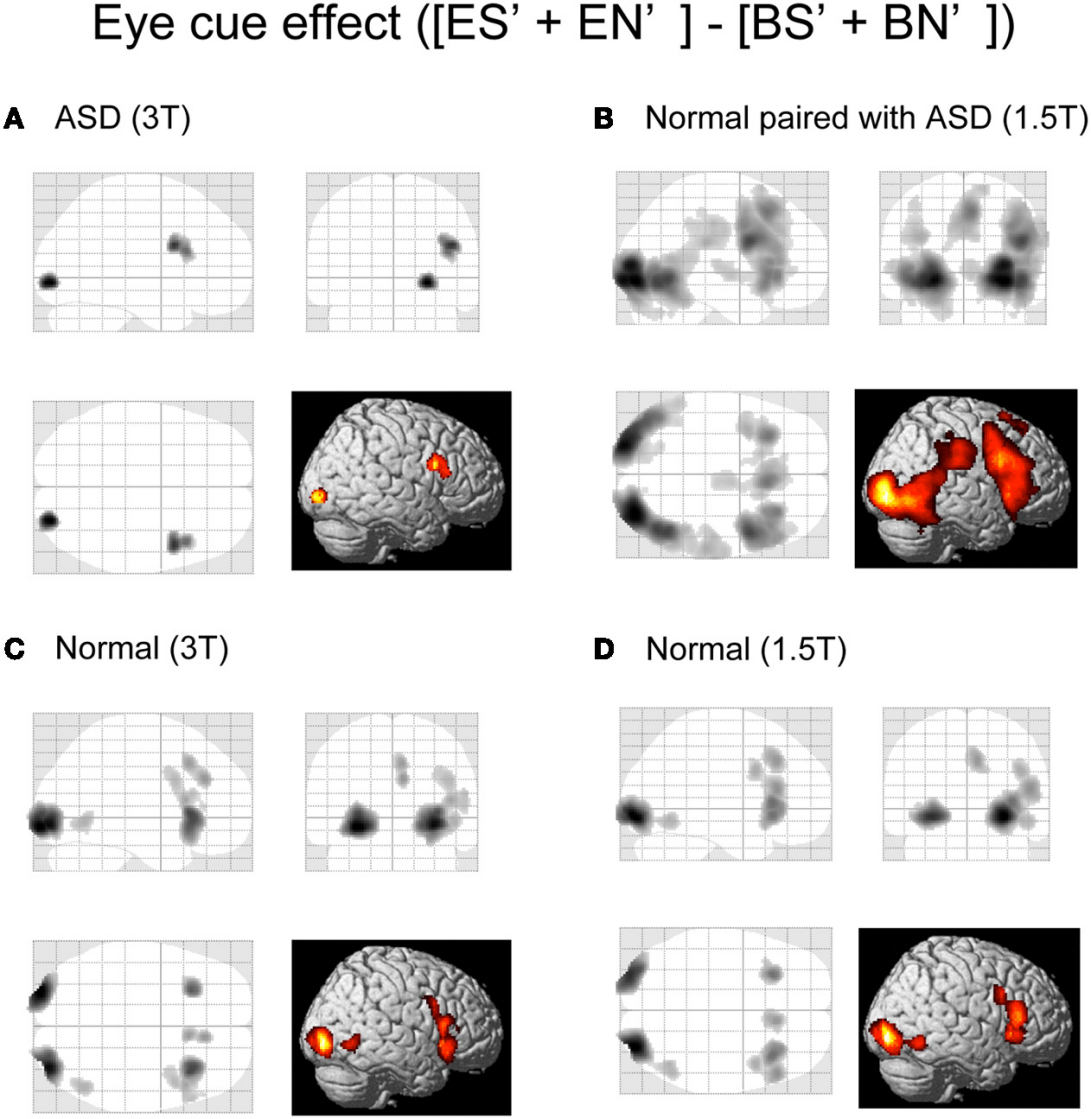
**Activation maps of the eye-cued effect.** Eye cue-related activities [(ES' + EN')–(BS' + BN')] are shown. **(A)** ASD participants in a 3T MRI scanner. **(B)** Normal participants paired with ASD participants in a 1.5T MRI scanner. **(C)** Normal participants paired with normal participants in a 3T MRI scanner. **(D)** Normal participants paired with normal participants in a 1.5T MRI scanner. The statistical threshold was *p* < 0.01 uncorrected for multiple comparisons at the peak level, and the cluster size was >50 voxels. Activation areas are shown on a glass brain in stereotaxic space with 3D information collapsed onto 2D sagittal, coronal, and transverse images, and a surface-rendered high-resolution MR image from the SPM template.

Initially, we examined the eye-cued effect [(ES' + EN')–(BS' + BN')], the shared-attention effect [(ES' + BS')–(EN' + BN')], and their interaction, in ASD participants, Normal participants paired with ASD participants, and the Normal groups from the previous study (Saito et al., 2010). The spatial extent of the activation of the eye-cued effect was reduced in the ASD group compared with the Normal groups (Figures 3A,C), whereas the activation was greater in the Normal paired with ASD group than in the Normal groups (Figures 3B,D). Specifically, the Normal control groups showed activation in the bilateral lateral occipital gyrus (LOG) including the OP, right middle temporal gyrus (MTG), posterior rostral medial frontal cortex (prMFC), and right IFG (Figures 3C,D). By contrast, the ASD group showed a smaller region of activity induced by the eye cue, which was observed in the right LOG and IFG (Figure 3A). The Normal paired with ASD group showed activation in the bilateral LOG including the OP extending to the human middle temporal complex (hMT+), MTG, right STS, the anterior portion of right inferior parietal lobe (IPL), prMFC, right middle frontal gyrus (MFG)/IFG, and bilateral insula (Figure 3B).

There was no statistically significant shared attention-related activity or interaction in the ASD and Normal paired with ASD groups.

**Figure 4:**
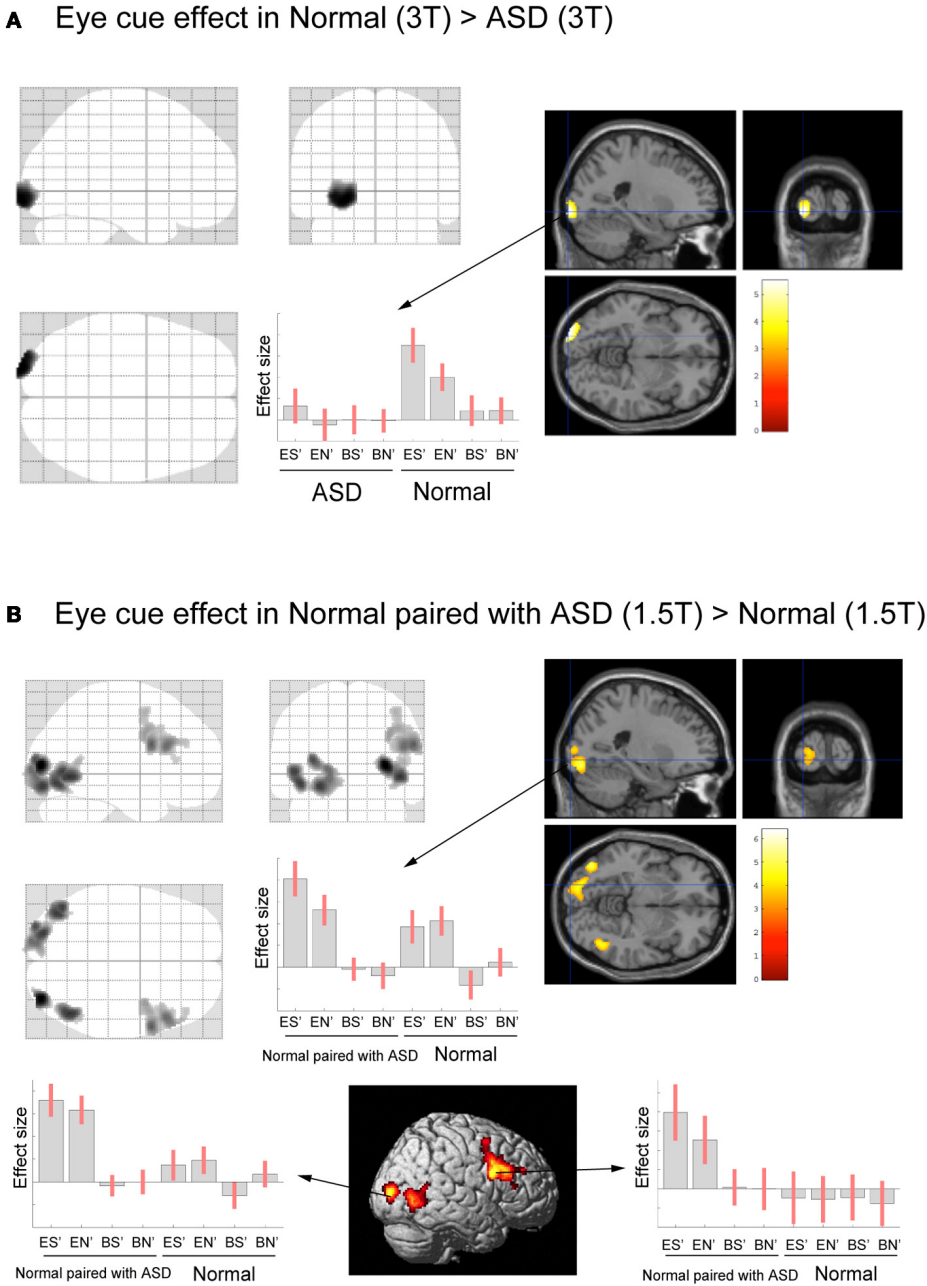
**Activation maps for direct comparison between ASD (3T) and Normal (3T), and Normal paired with ASD (1.5T) and Normal (1.5T).** The statistical threshold was *p* < 0.05 with an FWE correction at the cluster level for the entire brain. **(A)** Normal (3T) > ASD (3T) activation areas are shown on a glass brain (right), and on sagittal, coronal, and transverse T1-weighted SPM template images around the local maximum of the lateral occipital gyri (LOG). The effect size of the local maximum (*x* = −22, *y* = −100, *z* = −6) in the LOG is shown during each task condition. **(B)** Paired with ASD (1.5T) > Normal (1.5T) activation areas superimposed on a glass brain, section images around the left LOG, and a surface-rendered high-resolution MRI of the SPM template. The center part of the plot shows the effect size in the position described in A (*x* = −22, *y* = −100, *z* = −6) during each task condition. The bottom part of the plots shows the effect size of the local maximum in the right LOG (*x* = 32, *y* = −86, *z* = 8) and in the dorsal part of the IFG (*x* = 58, *y* = 10, *z* = 26) during each task condition, respectively.

Next, we conducted a direct comparison between ASD (or Normal paired with ASD) participants and the normal individuals who participated in our previous study. To eliminate any scanner effects, the ASD group was compared with the Normal group data from the 3T scanner, whereas the Normal paired with ASD group was compared with the Normal group data from the 1.5T scanner. The eye cue-related activity in the left LOG (in the OP) was reduced in the ASD group compared with the Normal groups (Figure 4A, Table 2A). There was no significant correlation between IQ and BOLD response of the OP in eye-cued conditions (IQ and ES, *r* = 0.202, *p* = 0.406; EN, *r* = 0.308, *p* = 0.200, respectively).

**Table 2:** **Direct comparison of Normal (3T) > ASD (3T) and Normal paired with ASD (1.5T) > Normal (1.5T)**.

**Task**	**Cluster level**					**Peak level**			
	***p*-value (FWE corr)**	**Cluster size**	**MNI coordinates**			***z*-value**	***p*-value (uncorr)**	**Side**	**Location**
			***x***	***y***	***z***				
**(A) NORMAL (3T) > ASD (3T) (REDUCED ACTIVATION IN ASD GROUP)**									
Eye cued	0.026	414	−22	−100	−6	5.23	<0.001	L	LOG
**(B) NORMAL PAIRED WITH ASD (1.5T) > NORMAL (1.5T) (GREATER ACTIVATION IN NORMAL PAIRED WITH ASD GROUP)**									
Eye cued	0.012	297	32	−86	8	5.99	<0.001	R	LOG
	0.001	488	46	−62	2	5.10	<0.001	R	LOG
	<0.001	1225	−24	−86	8	4.96	<0.001	L	LOG
			−46	−68	2	4.90	<0.001	L	LOG
	<0.001	1224	58	10	26	4.51	<0.001	R	IFG
			50	24	24	4.34	<0.001	R	IFG

Results of the random-effects analysis for the direct comparison between ASD (3T) and Normal (3T) **(A)** and between Normal paired with ASD (1.5T) and Normal (1.5T) **(B).** The statistical threshold was p < 0.05 with an FWE correction at the cluster level for the entire brain. ASD, autism spectrum disorder; FWE, family-wise error; IFG, inferior frontal gyrus; L, left hemisphere; LOG, lateral occipital gyri; MNI, Montréal Neurological Institute; R, right hemisphere.

In contrast, the Normal paired with ASD group (1.5T) showed greater eye-cued activity than the Normal groups (1.5T) in the bilateral LOG, including hMT+, and the dorsal portion of the right IFG (Figure 4B, Table 2B). The ball-cued effect did not statistically differ between the ASD (3T) and Normal (3T) groups, or between the Normal paired with ASD (1.5T) and Normal (1.5T) groups (data not shown), indicating that the difference of activation was specific to the eye-cued conditions.

### Inter-brain coherence

**Figure 5:**
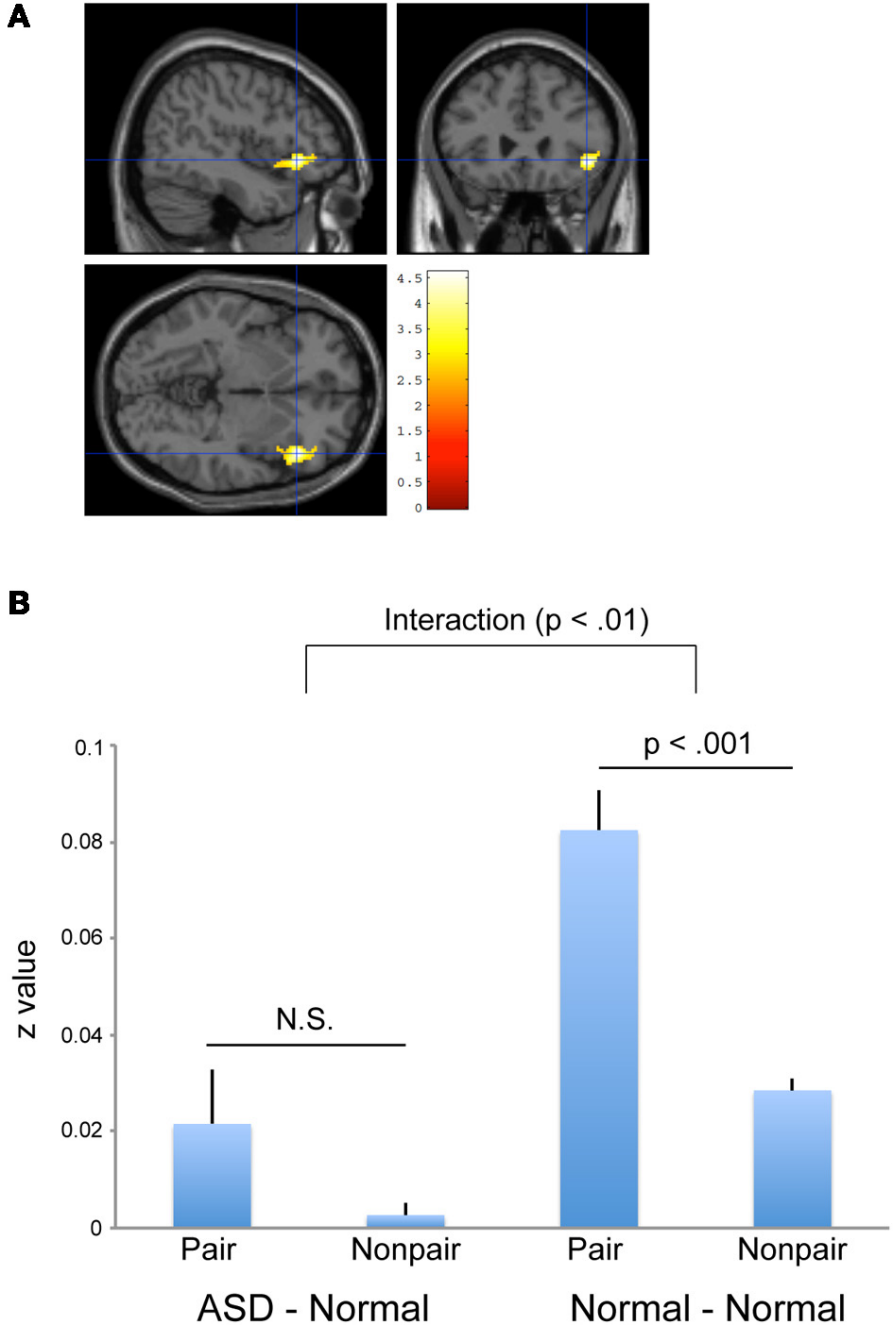
**Inter-individual correlation using residual data. (A)** Significant positive inter-individual correlation (Pair > Non-pair) in the Normal—-Normal experiment based on residual time-series data obtained by SPM8. The statistical threshold was *p* < 0.05 with an FWE correction at the cluster level for the entire brain. The area was superimposed on sagittal, coronal, and transverse T1-weighted SPM template images. **(B)** Between-subject correlations in the right IFG (*x* = 44, *y* = 26, *z* = −6) calculated with the residual data obtained by SPM8. An Experiment (ASD–Normal vs. Normal–Normal) × Pairing (Pair vs. Non-pair) interaction was observed (*p* < 0.01). A more prominent positive correlation was observed between the pair compared with the non-pair combinations in the Normal–Normal experiment (right side, *p* < 0.001), but not in the ASD–Normal experiment (left side, *p* = 0.502, N.S.). Error bars indicate the SEM.

A voxel-by-voxel analysis of “Pair” and “Non-pair” correlations between ASD–Normal did not show a statistically significant higher correlation in the “Pair” group across the whole brain. To confirm and compare the Normal–Normal pair results, a further ROI analysis was conducted using the residual time-series data from the ventral portion of the right IFG region that showed a high correlation (that is, Pair > Non-pair) in the Normal–Normal experiment. The residual time-series data were obtained using MarsBaR software (http://marsbar.sourceforge.net/). As we used a different version of SPM (i.e., SPM8) from the previous study (i.e., SPM5), the data from the earlier Normal–Normal experiment were re-analyzed (Figure 5A). After collecting the data, we calculated the correlation of the residual data for all possible combinations of the pairs (441 for ASD–Normal; 361 for Normal–Normal), and the correlation values were transformed to *z*-scores using Fisher's *r*-to-*z* transformation. The pairs were divided into two groups in each experiment: 21 pairs in which the two subjects participated simultaneously (Pair group) and 420 pairs in which they did not (Non-pair group) for the ASD–Normal experiment; and 19 pairs in which the two subjects participated simultaneously (Pair group) and 342 pairs in which they did not (Non-pair group) for the Normal–Normal experiment. We conducted a Two-Way ANOVA (Experiment × Pairing) using SPSS software (SPSS Inc., Chicago, IL, USA). The results showed an Experiment × Pairing interaction [*F*_(1, 798)_ = 4.892, *p* = 0.027] (Figure 5B). No statistically significant correlation difference was observed between Pair and Non-pair groups in ASD–Normal (*p* = 0.502, *post-hoc t*-test with Bonferroni correction), whereas a more prominent correlation was detected in the Pair compared with the Non-pair groups in the Normal–Normal experiment (*p* < 0.001, *post hoc t*-test with Bonferroni correction).

### Difference of right IFG intra-brain functional connectivity

**Figure 6:**
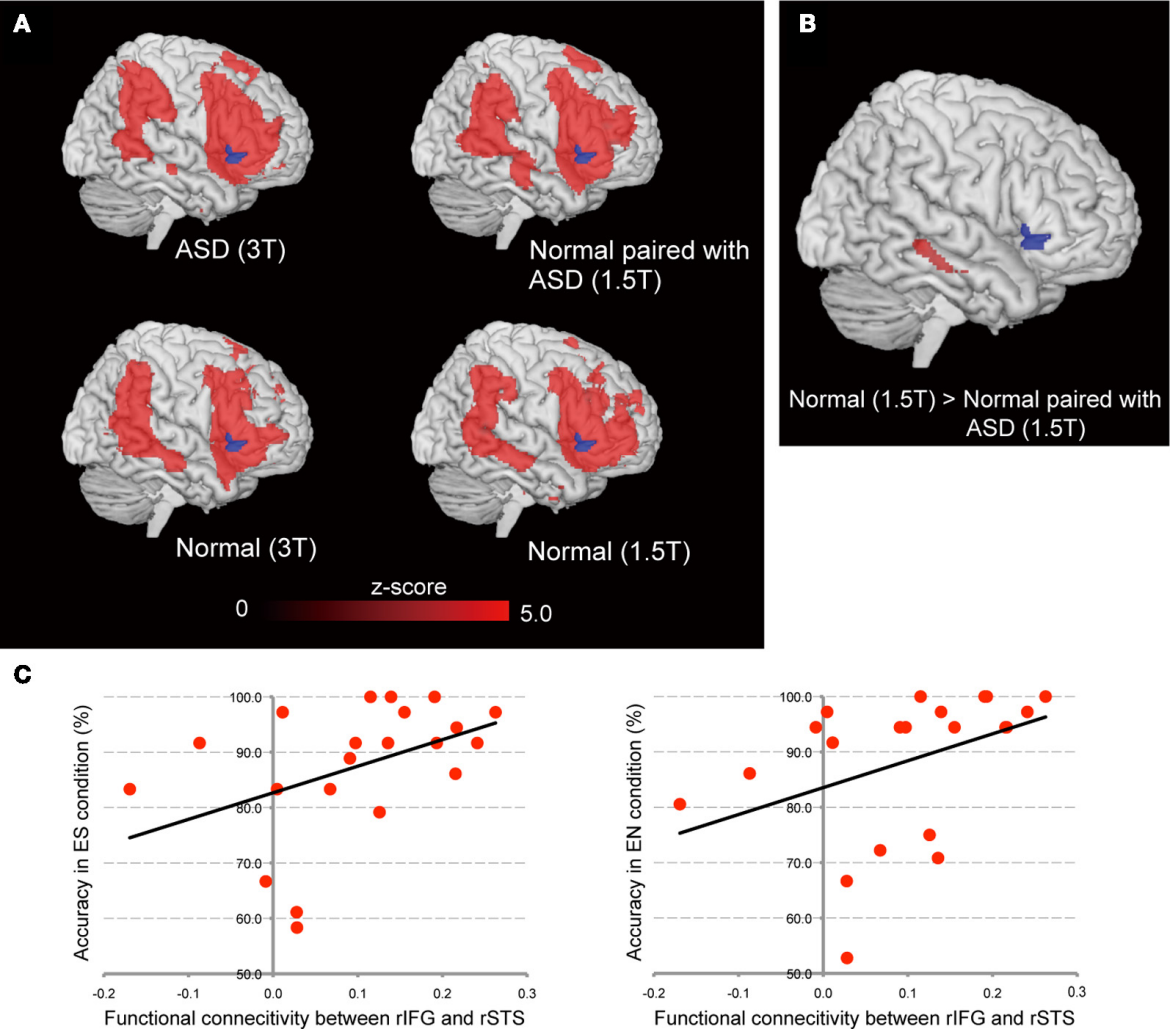
**Functional connectivity maps from the right IFG seed using the residual data. (A)** Results for the ASD group (upper left) and Normal paired with ASD group (upper right), and the Normal (3T) and Normal (1.5T) groups (lower left and right, respectively). The blue area denotes the seed region (right ventral IFG). Red regions showed statistically significant functional connectivity with the seed region. **(B)** Difference of connectivity strength between Normal (1.5T) and Normal paired with ASD (1.5T) groups. The statistical threshold was *p* < 0.05 with an FWE correction at the cluster level for the entire brain. **(C)** Correlation between the functional connectivity strength of right IFG–STS and accuracy in the ES (left side) and EN (right side) conditions, respectively, in the Normal paired with ASD group.

To explore which regions were involved in the common psychological ground network, we conducted functional connectivity analysis using the right ventral IFG as a seed region. To examine right IFG intra-brain functional connectivity between the ASD, Normal paired with ASD, and Normal groups, functional connectivity maps were compared between the ASD and Normal groups, and the Normal paired with ASD and Normal groups. To minimize the effect of the scanner, data from the same machine were compared [that is, ASD (3T) vs. Normal (3T), Normal paired with ASD (1.5T) vs. Normal (1.5T)]. The right ventral portion of the IFG was identified as a seed region based on the findings of the inter-individual correlation analysis. Initially, we identified the functional connectivity map in each group (Figure 6A). As expected, the right ventral IFG showed functional connectivity with lateral and medial frontal, parietal, and temporal regions. No statistically significant difference was observed between the ASD (3T) and Normal (3T) groups. By contrast, the functional connectivity between the right ventral IFG and right STS was significantly weaker in the Normal paired with ASD (1.5T) than the Normal (1.5T) group (Figure 6B). Because the accuracy in the eye-cued condition varied among individuals of the Normal paired with ASD group, we calculated the correlation between the connectivity strength and accuracy in the ES and EN conditions, respectively, and identified either a statistically significant positive correlation or a trend (Figure 6C).

## Discussion

### Performance

Individuals with ASD showed decreased accuracy in the eye-cued task compared with normal controls (Normal–Normal pairs). As performance on the ball-cued task was similar to that of the control groups, this deterioration was specific to the eye-cued condition. Performance was impaired not only in ASD participants, but also in their normal partners (Figure 2). Reduced accuracy was observed during the eye-cued condition in ASD–Normal but not Normal–Normal pairs (that is, there was a Group × Task interaction), suggesting that impaired performance was specific to the ASD–Normal pairs during the eye-cued tasks. As no performance differences were observed between the ASD–Normal and Normal–Normal pairs in the control ball-cued tasks, this finding indicates that this effect was specific to the gaze-exchange interaction between the ASD–Normal pairs. This effect might be caused by difficulties in making and keeping eye-contact in the ASD-Normal pairs, although we did not measure the difficulty in EC in the present study. EC in individuals with ASD is reportedly abnormal, and is not used to initiate joint attention (Sigman et al., 1986; Baron-Cohen, 1989, 1995). As gaze direction explicitly indicates the target of the attention, EC is regarded as mutual, shared attention with another person (Saito et al., 2010). Thus declined gaze fixation of ASD patient makes EC difficult for the normal partner.

Considering that performance of ES and EN relies on the change detection of the gaze from the EC condition, difficulty in EC may result in the deterioration of the eye-gaze detection of normal individuals when paired with ASD participants.

### Reduced eye-cued activity in ASD and greater activity in the normal paired with ASD group

Direct comparison between the ASD–Normal and Normal–Normal groups revealed reduced eye cue-related activity in the left OP in ASD individuals. To eliminate any scanner effect, we compared the ASD group to the results for normal participants in the 3T MRI scanner. Although the IQ of the normal participants was unavailable, this finding may not reflect the difference in IQ, because (1) IQ of the ASD group is within normal range (Table 1), and (2) there was no significant correlation between IQ and BOLD responses of the OP in ASD group (ES and EN). Nevertheless, we found decreased activity of the OP in ASD participants. This region is close to the recently reported kinetic occipital area (KO), which is known to be related to both shape- and motion-information processing (Orban et al., 1995; Dupont et al., 1997). This region is also involved in the visual processing of social stimuli. The OP is related to animacy perception (Morito et al., 2009), and is activated by eye-gaze cues in healthy adults (Tipper et al., 2008; Greene et al., 2009; Engell et al., 2010), and in typically-developing children and adolescents (Greene et al., 2011). Dalton et al. (2005) reported that ASD patients showed less gaze fixation on the eye areas of the visually presented static faces, and less activation in the bilateral OP. Thus, fewer fixation may result in less accuracy and weaker activation of the OP that is related to the early visual processing (Morito et al., 2009). Because of technical difficulty, we could not analyze the eye fixation during fMRI experiment in the present study. Quantitative analysis of EC will be necessary for future study. However, based on the present study, it is conceivable that the decreased eye-cued activity of the OP in ASD individuals may represent abnormal eye-gaze processing. The deficit is not specific to the RJA but related to the eye-cued processing *per se*. Considering that gaze following is essential for RJA, this finding is consistent with the neuro-developmental model of ASD which postulates the cascading effect of atypical RJA on later behavioral development (Mundy and Jarrold, 2010).

We also directly compared the normal individuals paired with ASD participants to those paired with normal partners. Again, to eliminate any scanner effect, we compared data from these groups acquired using the 1.5T MRI scanner. Normal participants paired with ASD participants showed greater activity in the bilateral occipital cortex, including the left OP, and the right dorsal portion of the IFG. The behavioral results showed that it was difficult for normal individuals to detect ASD individuals' eye gaze; enhanced activation in the visual cortex, including the OP, might therefore represent a higher workload to process eye gaze in individuals with an ASD partner compared to those with a normal partner.

As the right dorsal portion of the IFG did not show eye cue-specific activation in the normal subjects paired with normal subjects (Figure 4B), the enhanced activity in ASD partners might be related to non-specific factors, such as increased attentional demands.

### Inter-brain coherence between ASD and normal participants

Consistent with the behavioral results, inter-brain coherence in the right ventral IFG was significantly less prominent in the ASD–Normal pairs than the Normal–Normal pairs. Specifically, we observed an Experiment (ASD–Normal vs. Normal–Normal) × Pairing (Pair vs. Non-pair) interaction in the right IFG. The right ventral IFG is one of the key regions for social information processing. Passive viewing of averted eye movements activates the right IFG (Pelphrey et al., 2005). It is also related to the unconscious mimicry of the face (Leslie et al., 2004), and to self–other face distinction (Sugiura et al., 2006; Morita et al., 2008), suggesting a role in self–other interactions. Kosaka et al. (2010) showed that ASD participants had decreased volume of the right IFG, the size of which showed a negative correlation with AQ scores.

In our previous report, we suggested that the ventral portion of the right IFG is the site of the neural representation of the common psychological ground or shared intention mediated by EC (Saito et al., 2010). In this context, the weakened inter-brain synchronization of the right IFG in the ASD–Normal pairs might reflect a difficulty in integrating the self- and other-oriented attention.

### Intra-brain functional connectivity with right IFG

According to the parallel and distributed Process (PDP) model, IJA is represented by the anterior attentional system that yields self-perception (“where my eyes go, my own behavior follows”), while RJA represented by the posterior attentional system brings other-perception (“where other's eyes go, their behavior follows”), and these percepts are integrated (Mundy and Jarrold, 2010). From this view, IJA is associated with frontal-cortical activity whereas RJA is closely tied to parietal and temporal cortical processes (Mundy and Jarrold, 2010). In support of this notion, Redcay et al. (2012a) reported that the right IFG was activated by both RJA and IJA. Thus the neural inter-subject synchronization in the right IFG may represent the overlap of two distinct and parallel attentional system and integration of the self-other perception. To test this, we evaluated the intra-brain functional connectivity of right IFG. To explore this, functional connectivity analysis was conducted using residual time-series data with the right ventral IFG as a seed region. As both participants continuously gazed at each other as a baseline condition, eye-gaze processing can be represented as the intra-brain functional connectivity with the right IFG. The results showed that the brain activity in the lateral and medial part of the frontal cortex and parieto-temporal regions, including the temporo-parietal junction (TPJ) and STS, fluctuated coherently. This finding indicates neural synchronization mediated by EC may be related to the integration of anterior and posterior attentional system which represents IJA and RJA, respectively. As present study did not include the IJA component, involvement of IJA in the neural synchronization of right IFG and its attenuation in ASD–Normal pair is to be investigated in future studies.

There was no significant reduction in the intra-brain connectivity in the ASD group compared with the Normal group. This finding suggests that the poorer performance on joint-attention tasks in ASD individuals might not be due to right IFG dysfunction, but rather to the impaired early visual processing of eye gaze, which is represented by the reduced OP activation. However, we observed a statistically significant reduction in functional connectivity between the right IFG and the right anterior part of the STS in the normal participants paired with the ASD participants. Furthermore, the right STS–IFG functional connectivity in normal participants paired with ASD participants was positively correlated with their performance during the eye-cued joint-attention task. The right anterior STS is known to respond to direct gaze (Calder et al., 2002; Wicker et al., 2003), suggesting that EC facilitates the encoding of gaze direction in this region (Calder et al., 2007; Nummenmaa and Calder, 2009). This might suggest that the inter-individual synchronization of brain activity during EC is a prerequisite for joint attention to be achieved, at least in normal individuals. This finding suggests that the cause of the poorer performance in the joint-attention task differs between individuals with ASD and their normal partners. In the ASD group, impaired performance might be caused by dysfunctional early visual processing of eye gaze, as indicated by reduced activation in the OP. In the normal partners of ASD individuals, poorer performance might be caused by a lack of common psychological ground that is represented by the inter-subject coherence in the right ventral IFG, which is in turn mediated by the right anterior STS, which has a central role in gaze processing (Grosbras et al., 2005). The performance decline in the normal participants paired with ASD participants might be partly compensated for by enhanced early visual processing, as indicated by the increased eye cue-related activity of the early visual areas in this group compared with the control Normal–Normal pair participants.

Schematically, the postulated neural mechanism of mutual eye gaze processing is as follows. Initially, the eye-gaze signal is processed in the OP and LOC, and mediated by the right IFG. Eye-gaze shifts during joint attention are also processed in the OP and LOC, and mediated by the right anterior STS, where the gaze-movement signal from the visual areas and the context signal from the IFG are integrated. Dysfunction of the OP in ASD individuals might reduce joint attention-task performance, and also lead to unstable EC with their partners; this failure to establish EC is represented by decline of the inter-individual coherence of brain activity in the right ventral IFG, which in turn sends context signals to the right anterior STS for integration. This causes the decline of joint gaze-detection task performance in the normal individuals paired with ASD participants, despite the compensatory enhancement of early visual processing represented by the hyper-activation of the OP and LOC.

### Comparison with the previous study using live JA tasks

Using live interaction joint attention tasks, Redcay et al. (2012a,b) succeeded in depicting activation patterns related to IJA and RJA in both ASD group and normal control group. The dMPFC showed a reduced difference between joint attention conditions and control condition in the ASD, compared to that in the normal group. Redcay et al. (2012b) argued that dMPFC may play a role in both mutual engagement with a social partner (or dyadic attention) as well as sharing attention with another on an object or event (or triadic attention), both of which are critical to establishing joint attention. The left pSTS showed increased activation for the joint attention versus solo attention conditions in the normal control group, but not in the ASD group. Redcay et al. (2012b) interpreted this finding as an evidence of reduced selectivity of the response to social stimuli in ASD group.

As they used single fMRI setting, they did not explore the neural underpinning of the interaction of two persons during face-to-face communication through the eyes. In the present study, we adopted RJA-type task mainly focused on the two-persons' interaction. Critically, we measured the paired participants' brain activity to evaluate the neural synchronization. We found that the activity of the OP related to eye-cue was reduced in ASD group. ASD–Normal pair diminished the eye-contact related synchronization in the right IFG. These differences are not RJA specific. Therefore these findings are related to the ASD's dysfunction of the elementary component of RJA, that is, eye-gaze processing as biological motion, and neural synchronization during EC.

## Conclusion

To our knowledge, this is the first study to demonstrate the neural correlates of direct, real-time interaction between individuals with ASD and normal subjects. The findings suggest that the impairment of joint attention in ASD is related to hypo-function of early visual processing and difficulty in understanding shared intention through EC, which is represented by reduced inter-subject synchronization of cortical regions including the right IFG.

### Conflict of interest statement

The authors declare that the research was conducted in the absence of any commercial or financial relationships that could be construed as a potential conflict of interest.
